# Moderate size infantile haemangioma of the neck – conservative or surgical treatment? : a case report

**DOI:** 10.1186/1752-1947-2-52

**Published:** 2008-02-19

**Authors:** Abdulzahra Hussain, Hind Mahmood, Hussein Almusawy

**Affiliations:** 1 General surgery department, Princess Royal University Hospital, Kent, UK; 2 General surgery department, Alburaihy hospital, Taiz, Yemen

## Abstract

**Introduction:**

Infantile haemangioma is the commonest benign tumour in infancy. While the management of the majority of small haemangiomas consists of simply watching or steroid treatment, giant and moderate size infantile haemangiomas are challenging problems, especially in health systems with limited resources in developing countries.

**Case presentation:**

A one-year old boy was presented to us by his parents with a moderate size haemangioma on the posterior triangle of the left side of the neck. Clinical assessment and radiological examinations were helpful in confirming the diagnosis. Surgical excision was performed successfully without major morbidity. Partial necrosis of the skin flap developed shortly after the operation but healing was complete in eight weeks. There was no residual problem on review five years after the operation.

**Conclusion:**

Early surgical excision of a moderate size infantile haemangioma may be justified especially when there is difficulty of follow-up, which can be a common problem in developing countries. This approach will prevent growth deformation, impact on nearby vital organs and psychological problems.

## Introduction

Infantile haemangioma (IH) is the commonest benign tumour of infancy [1]. Knowledge about the differential diagnosis can enable clinicians to detect haemangiomas that may lead to complications that will necessitate a multidisciplinary approach [2]. Although the majority of patients are treated conservatively, there is a need for surgical resection in certain cases depending on the size and site of the lesion and parental preference for a specific intervention. However, patients do respond very well to the wait and see policy and to steroid therapy.

## Case presentation

A one-year-old boy was presented by his parents to the outpatient clinic at Alburaihy Hospital in Taiz in Yemen in October 1999. The family described a progressive enlargement of a lump on the left side of the neck of an otherwise healthy infant.

**Figure 1:**
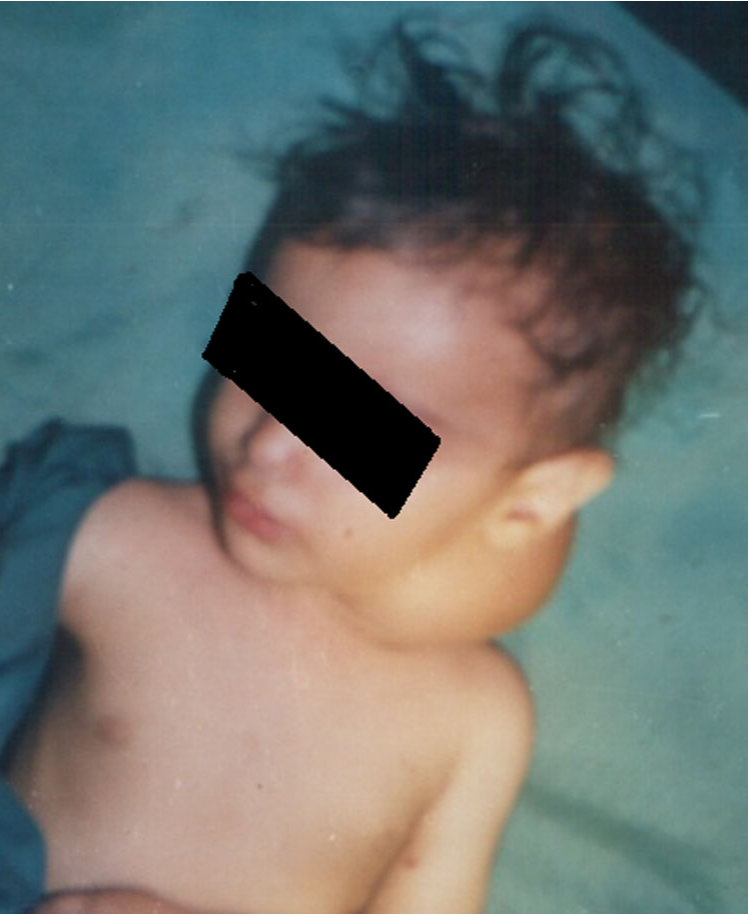
Anterolateral preoperative view.

**Figure 2:**
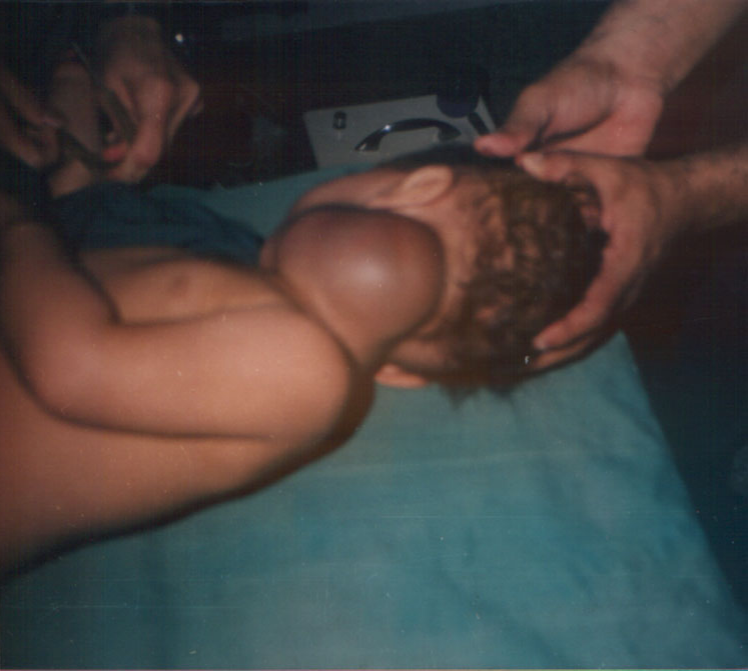
Posterolateral preoperative view.

Examination confirmed a 7 × 10 cm vascular tumour at the posterior triangle of the neck on the left side(see figure 1, 2). Full blood count, biochemistry, chest and neck X-rays were reported as normal apart from the soft tissue mass on the left side of the neck.

Ultrasound examination confirmed the cystic nature of the mass and a provisional diagnosis of infantile haemangioma was made. The wait and see policy, steroid and surgical options were explained to the parents and they chose surgery.

Successful resection of the haemangioma was performed through an elliptical incision parallel to the posterior edge of the sternomastoid muscle.

**Figure 3:**
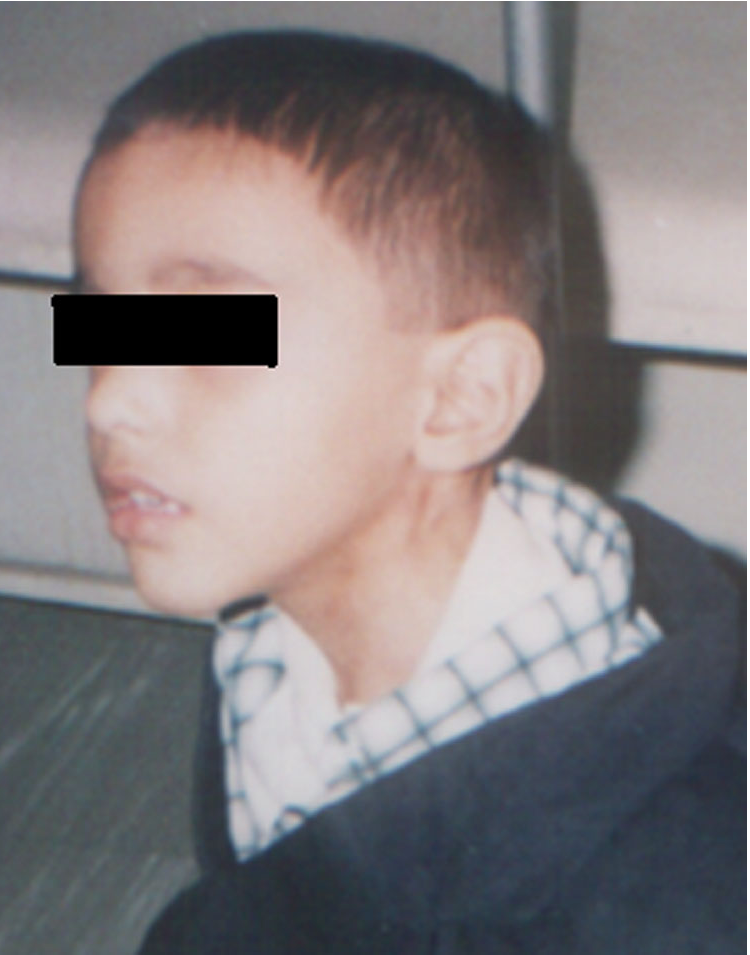
Five years after the operation.

Unfortunately, the operation was complicated by necrosis of the skin at the closure site. This was treated conservatively by wound dressings. No other morbidities were reported. During the subsequent follow-up, the wound healed completely in two months. At review after five years, the child was healthy and had no residual problems (see figure 3).

## Discussion

In developing countries, a lack of expertise is a key factor in many health issues including the management of complex vascular lesions of the head and neck. The approach to this lesion could be conservative or surgical, depending on certain factors including the age of the patient, and the size and site of the lesion. In order to prevent possible irreversible pressure complications, early diagnosis is important to manage vascular malformations correctly because of their distinct differences in morbidity, prognosis and treatment [3].

On the other hand, the social factors and associated problems of health settings in developing countries, including difficulties with follow up, the desire of the parents for immediate cure of the problem, and the variable success rates of the different conservative treatment modalities, may lead to a preference towards surgical excision. This may be the best option treatment for some but of course not all cases of IH.

Infantile haemangioma is a common problem. In a study of 900 patients, IH accounted for 25% of soft tissue tumors [4].

There is female predisposition especially for syndromes associated with haemangioma [5]. Most hemangiomas are easily diagnosed without any additional diagnostic tests such as magnetic resonance imaging MRI and the natural course of immature haemangiomas in infants is well known. The characteristic MRI findings include a focal, lobulated soft-tissue mass that is isointense relative to muscle on T1-weighted images and hyperintense on T2-weighted images. It has diffuse and homogenous contrast enhancement and dilated feeding and draining vessels within and around the mass [6]. Ultrasound examination US may be used during the initial assessment or in place of MRI if it is unavailable. High vessel density and high peak arterial Doppler shift can be used to distinguish haemangiomas from other soft-tissue masses with high sensitivity and specificity [7].

Since most of these lesions remain asymptomatic and resolve spontaneously, conservative management is generally the rule [8,9]. Nevertheless, the treatment options include surgical and non-surgical methods. Systemic steroid therapy may be indicated in IH and the reported success is documented [10,11]. Corticosteroid treatment, although recognized worldwide as a treatment of problematic haemangiomas cannot always control the growth of alarming haemangiomas [12]. In these cases surgical excision may be indicated.

Furthermore, for patients with severe problems, giant growth, and local complications surgical treatment can be a wise decision [13]. Early surgery can be proposed in order to avoid definitive deformation or growth impairment of adjacent structures. It should be performed before school age and before the occurrence of psychological difficulties [14].

The surgeon should be well prepared for intra-operative and post-operative complications of excision of large neck haemangiomas. Iatrogenic injury to adjacent vital structures, such as major vessels; nerves, airway, and gastrointestinal tract (especially with deeper lesions), are the most important morbidities. Skin and soft tissue complications are less risky and can be managed successfully in the majority of cases. Skin necrosis was reported in our patient. This was anticipated because of the size of the lesion and the adherence of the skin to the hemangioma. It was managed by frequent dressings and outpatient assessment. No plastic procedure was performed because complete healing was ensured two months after the operation.

## Conclusion

Early surgical resection of infantile haemangiomas can be a successful management option, especially for giant lesions. This approach will prevent growth deformation, impact on nearby vital organs and psychological problems.

## Abbreviations

Infantile haemangioma (IH); Ultrasound examination (US); Magnetic resonance imaging (MRI)

## Competing interests

The authors declare that they have no competing interests. The authors confirm that there are no financial competing interests and no non-financial competing interests that may cause embarrassment were they to become public after the publication of the manuscript.

## Authors' contributions

HA carried out the figures formatting, participated in the sequence alignment. HM participated in the sequence alignment. AH drafted the article and conceived the study, and participated in its design and coordination. All authors read and approved the final manuscript.

## Consent

Written informed consent was obtained from the patient's parents for publication of this case report and accompanying images. A copy of the written consent is available for review by the Editor-in-Chief of this journal.
